# *In-operando* FTIR study of ligand-linked Pt nanoparticle networks employed as catalysts in hydrogen gas micro sensors†

**DOI:** 10.1039/d3na00955f

**Published:** 2024-01-31

**Authors:** Daniel Loof, Oliver Thüringer, Volkmar Zielasek, Anmona Shabnam Pranti, Walter Lang, Marcus Bäumer

**Affiliations:** a University of Bremen, Institute of Applied and Physical Chemistry Leobener Str. 6 D-28359 Bremen Germany zielasek@uni-bremen.de; b University of Bremen, Institute for Microsensors, Actuators and Systems (IMSAS) Otto-Hahn-Allee 1 D-28359 Bremen Germany

## Abstract

Microporous networks of Pt nanoparticles (NP) interlinked by aromatic diamines have recently shown prospects of application as hydrogen combustion catalysts in H_2_ gas microsensors. In particular with respect to long-term sensor performance, they outperformed plain Pt NP as catalysts. In this paper, electron microscopy and Fourier transform infrared (FTIR) spectroscopy data on the stability of *p*-phenylene diamine (PDA) and of the PDA-linked Pt NP network structure during catalyst activation and long-term sensor operation at elevated temperature (up to 120–180 °C) will be presented. For the first time, all data were collected directly from microsensor catalysts, and FTIR was performed *in operando*, *i.e.*, during activation and sensor operation. While the data confirm high long-term catalyst activity far superior to that of plain Pt NP over 5 days of testing, they reveal that PDA fully decomposed during long-term sensor operation and that the network of discrete Pt nanoparticles changed to a sponge-like Pt nanostructure already during catalyst activation. These findings are at variance with previous work which assumed that stability of the PDA-linked Pt NP network is prerequisite for catalyst stability and performance.

## Abstract 2

Pt nanoparticles linked by aromatic diamines make superior combustion catalysts in H_2_ microsensors. Their long-term performance is probably based on diamine decomposition and formation of a sponge-like Pt nanostructure during catalyst activation.

## Graphical abstract

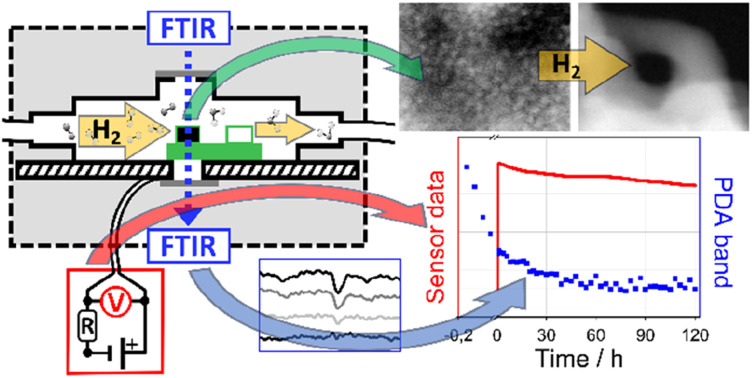

## 1. Introduction

Three-dimensional, microporous networks of Pt nanoparticles (NP) linked by diamine molecules recently turned out to be promising candidates for application as catalysts in H_2_ microsensors.^1–6^ Such networks have been synthesized from Pt NP with diameters of less than 2 nm and a selection of cycloaliphatic and various aromatic diamines, the choice of which allowed for tuning the average inter-particle distance within the networks in the range 1–2 nm.^4^ Designed to offer a high spatial density of Pt surface sites, these novel materials generate a significant heat tone from catalytic combustion of even minute amounts of hydrogen in air which can be exploited, *e.g.*, by thermoelectrical sensor designs. The synthesis of the NP networks can be performed directly on the sensor, simply by co-deposition of Pt NP in suspension and diamine ligands in solution.

Compared to other detection principles, thermoelectrical sensors typically stand out with respect to low power consumption and low operating temperatures.^7^ The microsensors investigated in the present study can detect down to 10 ppm hydrogen in synthetic air with a response time of 0.65 s, show a linear dependence on hydrogen concentration and no cross-sensitivity to other combustible gases such as methane, ethane or ethanol.^8^ Morsbach *et al.* were first to not only demonstrate high sensitivity and fast response of H_2_ microsensors based on Pt NP networks linked by *p*-phenylenediamine (PDA), but also show long-term stability of the catalyst performance, far superior to that of plain Pt NP.^3^ It was generally assumed that maintenance of the catalyst structure, *i.e.*, a ligand-linked network of discrete Pt NP, was key to the catalyst stability and that it was owed to thermal and chemical stability of the aromatic diamine ligands and their Pt bonds at sensor operating conditions, *i.e.*, typically at temperatures of 70–120 °C and with up to 1,5 vol% H_2_ in air.^1–3^

While these assumptions were supported by a thermogravimetric analysis which showed thermally induced decomposition of various diamines setting in only above 130 °C,^4^ a more recent temperature-programmed desorption (TPD) study of PDA-linked Pt NP networks (Pt-PDA) in vacuum indicated that in the presence of hydrogen, Pt-catalyzed decomposition of PDA might deteriorate its thermal stability.^6^ Furthermore, inspection of Pt-PDA catalysts by scanning electron microscopy (SEM) before and after long-term sensor operation revealed significant changes of their morphology on the micrometer scale.^5^ Microscopic evidence for the persistence of the ligand-linked Pt NP network structure and the intactness of the PDA ligands during long-term sensor operation has been missing, so far.

The experimental study presented in the following provides the first unambiguous insight into the effects of sensor operation on the structure of diamine-linked Pt NP networks on the nanometer scale. Electron microscopy and Fourier-transform infrared (FTIR) spectroscopy were employed to gain information on the catalyst morphology and the molecular stability of the diamine ligands as well. Besides effects of sensor operation, the present study also brings effects of catalyst activation into view. Similar to other catalysts, diamine-linked Pt NP networks must undergo a brief thermal treatment after synthesis in order to achieve maximum catalytic activity during sensor operation. So far, it was assumed that the activation procedure merely removed solvent residues from the Pt-PDA synthesis.^3^

To avoid any ambiguities of model studies which rely on, *e.g.*, excessive amounts of catalyst material or substitute substrates for the sake of experimental accessibility, all data presented below were directly collected from Pt-PDA catalysts synthesized on hydrogen gas microsensor chips for application. Furthermore, FTIR spectra were recorded *in operando*, *i.e.*, during sensor activation or operation, in order to identify any correlations between sensor performance and the molecular structure of the organic links in the Pt NP network. For that purpose, a small reactor module was constructed that allowed for *in operando* FTIR measurements in the evacuated sample compartment of a commercial spectrometer, as will be detailed in the experimental section. Challenges met by its design are, among others, the provision of full sensor operability (gas flow at ambient pressure, control of operating temperature, sensor signal detection), IR transparency of the reactor, and sufficient sensitivity despite merely microgram amounts of catalyst on a surface area of less than 0.5 mm^2^.

The experimental findings of the present study disprove major presumptions of previous work with respect to the stability of PDA-linked Pt NP network catalysts in H_2_ microsensors. As will be demonstrated in the following, Pt-PDA catalysts undergo severe structural changes on the nanometer scale already during activation, and the PDA ligands slowly but fully decompose during sensor operation. Nevertheless, Pt-PDA based catalysts maintain a fairly stable and high activity for hydrogen combustion, far superior to that of plain Pt NP catalysts. The data indicate the transformation of Pt-PDA into a thermally stable, mesoporous, sponge-like Pt nanostructure, probably stabilized by minute amounts of carbon residues, that could be of interest for niche applications in catalysis in its own right.

## 2. Experimental section

### 2.1. Materials synthesis and sample preparation

**Fig. 1:**
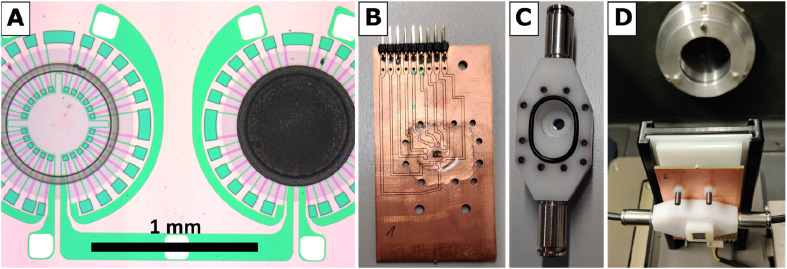
(A) Sensor chip detail showing reference membrane (left) and membrane with Pt-PDA catalyst (right) surrounded by thermopiles and heaters, (B) PCB with glued-on and electrically bonded sensor chip in the center, (C) reactor module housing, (D) reactor module assembled and aligned with the IR transparent Si windows in the beam path of the FTIR.

The hydrogen gas microsensor samples used in this study were based on an optimized and time-tested sensor chip design prepared by microtechnology from a commercial silicon wafer. The design features two identical circular SiN membranes (diameter 900 μm, thickness 500 nm), each surrounded by a WTi heater and a series of 24 thermocouples as thermopile (Fig. 1A). The reader is referred to ref. 5 and 9 for details of the sensor chip preparation.

For the synthesis of PDA-linked Pt NP networks, H_2_PtCl_6_·*x*H_2_O (ChemPur, Pt-wt.% 40), ethylene glycol (VWR, 99.7%), sodium hydroxide (VWR, 99.3%), hydrochloric acid (CHEMSOLUTE), cyclohexanone (VWR, 100.0%), and *p*-phenylenediamine (PDA) (Alfa Aesar, 97.0%) were used as purchased.

First, Pt nanoparticles were synthesized by the polyol process first described by Wang *et al.*^10^ Thereby, colloids of surfactant-free Pt NP in cyclohexanone were obtained with a Pt atom concentration of 123 mM (24 g L^−1^ Pt). For details of the NP synthesis and preparation steps the reader is referred to ref. 4.

Afterwards, catalytic layers of PDA-linked Pt NP were synthesized directly on one of the frangible SiN membranes of the sensor by subsequently depositing droplets of fresh PDA solution in cyclohexanone and of the surfactant-free Pt NP, freshly redispersed in cyclohexanone, *via* two piezo-driven microdispensers (microdrop Technologies GmbH). The PDA solution was prepared in the same concentration (123 mM) as the Pt colloid and both were applied in an equimolar ratio, yielding a total amount of about 1.4 μg of catalyst on the sensor after drying. Further details on the deposition process can be found in the ESI.† For comparison, in some experiments benzidine (BEN) (Sigma-Aldrich, 98.0%), 1,5-diaminonaphthalene (DAN) (TCI Europe, 98.0%), 4,4′′-diamino-*p*-terphenyl (DATER) (TCI Europe, 98.0%), or *trans*-1,4-diaminocyclohexane (DACH) (TCI Europe, 98.0%) were used instead of PDA for the synthesis of Pt NP networks. Prior to catalytic experiments, all sensor chip samples were stored under ambient conditions for a few days.

### 2.2. Reactor module for *in-operando* FTIR spectroscopy

FTIR spectra were recorded using a Vertex 80v spectrometer by Bruker Optics in transmission mode at a resolution of 4 cm^−1^. The spectrometer, equipped with a MIR source and a KBr beam splitter, was operated by the software OPUS 6.5. To improve the signal-to-noise ratio, each spectrum was averaged over 128 scans, corresponding to an acquisition time of 2 min per spectrum, and the sample compartment was evacuated to a base pressure below 300 Pa. Evaluation of FTIR spectra was facilitated by the software Spectragryph v1.2.14 (by Dr Friedrich Menges, non-commercial version).

For FTIR spectroscopy at the Pt NP catalyst of a hydrogen gas microsensor chip *in operando*, *i.e.*, during exposure of the sensor to a hydrogen-containing atmosphere and recording of a sensor signal, a small, leak-tight reactor module was constructed and assembled within the sample compartment of the spectrometer. The reactor module allowed for the IR beam of the spectrometer to selectively pass through the sensor membrane covered with the Pt NP catalyst while it provided the sensor with electrical leads to the WTi heaters and exposure to a continuous flow of 1.5 vol.-% H_2_ in synthetic air (Linde). Printed circuit boards (PCB) sized 40 × 70 mm^2^ were used as base plates of the module (*cf.*Fig. 1B). Each sensor chip was carefully positioned above a small center hole (diameter ∼1 mm) in a new PCB, glued thereon by epoxy resin, and subsequently bonded by thin aluminum wires to the PCB to establish electrical connections to the WTi heaters. Thereafter, the sensor chip was covered by a housing milled from inert polyoxymethylene (*cf.*Fig. 1C) that was pressed by screws onto the PCB. A Viton seal, fixed in place by a groove in the bottom of the housing, ensured a leak-tight connection between the housing and the PCB (The surface of the PCB was smoothened by a thin layer of epoxy resin to ensure a leak tight seal). The housing featured a small hole (diameter ∼1 mm) in-line with the center hole in the PCB underneath the sensor chip. Both holes were sealed gas-tight by glued-on (epoxy resin) windows made of silicon wafer dies which, together, exhibited an IR transparency of ∼25% in total. Note that also the SiN sensor membranes carrying the catalytic layer and being positioned right between the holes of PCB and housing were transparent for IR radiation. Fig. 1D shows a reactor module assembled in the sample compartment of the FTIR spectrometer and aligned with its Si windows in the IR beam path. To allow for a controlled flow of gas through the reactor module, the housing was equipped with two plug-in pneumatic connectors (Festo) which accepted pneumatic tubing made of polyurethane with 4 mm outer diameter (Festo). In addition, the cover of the sample compartment of the FTIR spectrometer was furnished with pneumatic and electrical feedthroughs so that the reactor module could be operated from the outside while the sample compartment was evacuated.

**Fig. 2:**
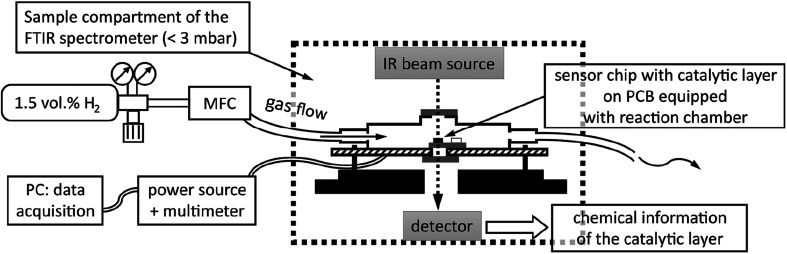
Schematic of experimental setup for catalytic hydrogen gas sensing with *in-operando* FTIR spectroscopy.

Fig. 2 is a schematic of the experimental setup, showing the reactor module in cross-section within the sample compartment (indicated by dashed lines). Depicted are the reactor housing on top of a PCB and thereon a sensor chip positioned so that the IR beam of the spectrometer passes through the Si windows of the module and that of the sensor membrane covered by the catalytic layer. The gas flow through the reactor was adjusted to 20 sccm for all experiments by a mass flow controller (MFC, EL-FLOW, Bronkhorst HI-TEC, max flowrate of 100 sccm) which was calibrated *via* a bubble flowmeter.

### 2.3. Hydrogen gas sensor operation

The standard operation principle of the hydrogen gas micro sensors used in this study is conversion of the heat tone of the exothermic hydrogen oxidation catalyzed by the Pt NP into an electrical signal generated by the thermopile which surrounds the SiN membrane covered by the catalytic layer (right side in Fig. 1A).^1,11,12^ To compensate for errors that may result from fluctuations of the ambient conditions, electrical measurements are performed in comparison to an identical, nearby membrane without catalyst (left side in Fig. 1A). The operating temperature of the sensor can be adjusted by electrical current through the WTi heaters of both membranes.

Note that in addition to a comparison of the thermoelectrical signals of the thermopiles of both membranes, also a comparison of the electrical resistances of both heaters surrounding the membranes allows for detecting any heat tone from hydrogen combustion because the electrical resistivity of WTi increases with temperature (thermoresistive sensor mode). To reduce the number of electrical feedthroughs required for the reactor module, only the thermoresistive sensor mode was used in this study, *i.e.*, electrical leads were needed only to the WTi heaters of both membranes. For sensor operation, both heaters were connected in series to an adjustable DC power supply, the voltage of which (*U*_0_) was set to adjust the desired idle operating temperature of the sensor as detailed below. During sensor operation, *U*_0_ was kept constant and the voltage drops across the heater of the sensor membrane with catalytic layer (*U*) and across the heater of the reference membrane (*U*_ref_) were continuously measured and automatically recorded at a time interval of 30 s *via* an AD converter and with the help of a National Instruments LabView script. In the series circuit, according to Ohm's law Δ*U* = *U* − *U*_ref_ is proportional to the difference of the electrical resistances of both heaters (*R* − *R*_ref_). Therefore, Δ*U* was taken as thermoresistive sensor output. (Small variations of the idle electrical resistances of both heaters typically led to small offsets in the range of a few 10 mV between *U* and *U*_ref_ even without any hydrogen present. This offset at idle conditions was subtracted from Δ*U* during subsequent operation of the respective sensor chip.)

To estimate the idle operating temperature adjusted by *U*_0_, the dependence on temperature of the electrical resistance *R* of the WTi heater surrounding the catalytic membrane was employed. It had been recorded *via* infrared pyrometry as fairly linear in previous studies and in accordance with the following equation:^9^*R*(*T*) = *R*(RT) × (1 + 3.64 × 10^−4^ K^−1^ × (*T* − RT))here, *R*(*T*) and *R*(RT) is the resistance of the heater at temperature *T* and at room temperature (RT), respectively. *R*(RT) was directly measured by a multimeter. To experimentally determine *R*(*T*) for a given *U*_0_ from voltage measurements, a constant electrical resistor of 100 Ω kept at room temperature (RT = 25 °C) was added as reference to the series circuit of both WTi heaters. The power supply was then adjusted so that *U* + *U*_ref_ corresponded to the desired *U*_0_ and the additional voltage drop across the 100 Ω resistor (*U*_100 Ω_) was measured by a multimeter. According to Ohm's law, the ratio of the measured voltage drops across the heater and across the 100 Ω resistor, respectively, corresponds to the ratio *R*(*T*)/100 Ω so that *R*(*T*) = 100 Ω × *U*/*U*_100 Ω_. The corresponding membrane temperature *T* was then derived from the equation above. In this study, *U*_0_ = *U* + *U*_ref_ was set to 7.00 V during sensor operation in order to heat the catalyst to an idle operation temperature of 70 °C.

### 2.4. Activation of the catalyst

Freshly prepared layers of PDA-linked Pt NP needed to be activated before they showed any catalytic activity for hydrogen combustion at an idle operation temperature of 70 °C. To activate the catalyst, the temperature of both sensor membranes was first increased to about 150 °C by setting *U*_0_ to 12.50 V. Then H_2_ in synthetic air was streamed through the reactor module as detailed above. Eventually, a temperature increase of the membrane with the catalytic layer was observed and indicated the onset of exothermic H_2_ oxidation catalyzed by the Pt NP. The temperature increase Δ*T* was quantified with the help of the equation above from the ratio *U*/*U*_ref_ which was taken as estimate for *R*(150 °C + Δ*T*)/*R*(150 °C).

The sensor activation was performed until the temperature of the catalytic membrane reached approx. 180 °C which usually required several minutes. Thereafter, the gas flow through the reactor was interrupted and *U*_0_ was reduced to 7.00 V. After activation, the catalytic H_2_ oxidation reaction started immediately when the H_2_ stream was reapplied to the sensor chip, as evidenced by a rapid temperature increase of the catalytic membrane from 70 °C to 100–110 °C. Within minutes, this increase leveled out at a maximum membrane temperature of *T*_max_ = 120 °C during continuous sensor operation.

### 2.5. Electron microscopy

For acquiring scanning electron microscopy (SEM) images, a Zeiss Auriga 40 field emission microscope equipped with a Gemini column and a SE2 detector (Everhart-Thornley) was used. The SEM was operated at a primary electron energy of 3 keV and controlled by the Smart-SEM V06.00 software.

For energy-dispersive X-ray spectroscopy (EDX) mapping, a Jeol JSM-6510 SEM (with tungsten hairpin cathode) operated at a primary electron energy of 4 keV and equipped with a Bruker Nano XFlash Detector 410 M (for EDX) was available. Data acquisition was performed with the help of the SEM Control User Interface version 3.02 as well as the Bruker Esprit 1.9 software.

For scanning transmission electron microscopy (STEM), a FEI Tecnai S TWIN microscope equipped with a Schottky field emission gun (FEG) and a high-angle annular dark-field (HAADF)-STEM detector was employed. The TEM was operated at a primary electron energy of 200 keV. An EDAX r-TEM detector allowed for elemental analysis *via* EDX.

Since complete sensors could not be inserted into the TEM column, sensor membranes with ligand-linked Pt NP network samples on top were broken into small chips that could be supported by standard carbon-coated Cu-TEM grids. For membrane chips which landed on the TEM grids with the catalytic layer facing towards the cathode of the TEM column, the quality of STEM images of the Pt NP appeared not to be compromised by the SiN substrate.

EDX spectra in STEM were acquired with a dwell time of 120–240 s. Quantification of the elemental composition (C, Pt, Si, N, O, and Cl) from these spectra was performed with the FEI TIA software version 5.0 (Thermo Fisher Scientific).

## 3. Results and discussion

**Fig. 3:**
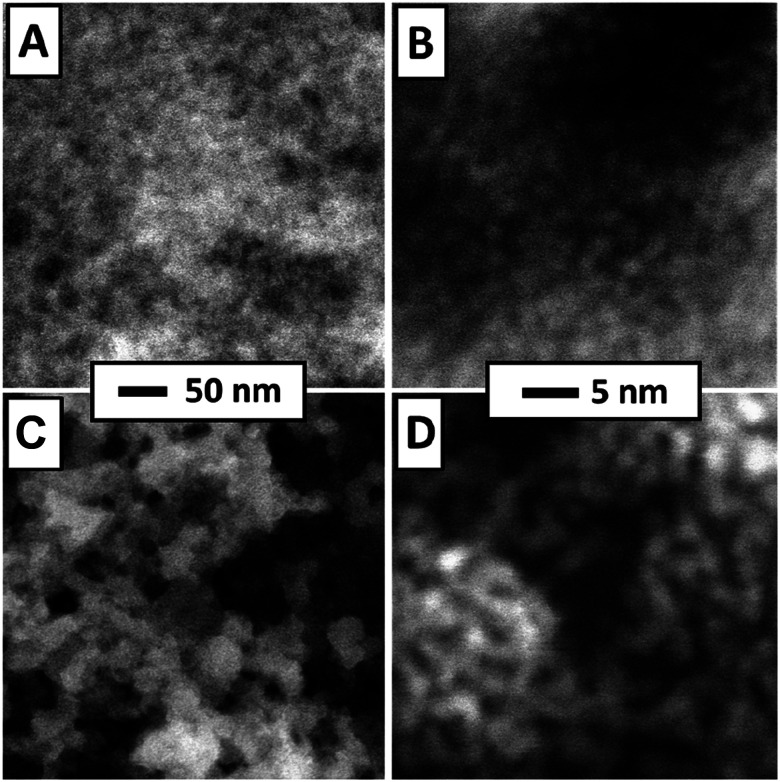
STEM of Pt-PDA (A) and (B) and pure Pt NP (C) and (D) on SiN sensor membrane. Overview images (left) display 400 nm × 400 nm of sample area, higher magnification (right) 35 nm × 35 nm.

STEM images of a freshly prepared catalytic layer, synthesized from PDA and Pt NP directly on a SiN sensor membrane, are presented in Fig. 3A and B. Such data have not been available before. Fig. 3A shows a fairly homogenous distribution of Pt within the layer (the PDA ligands are not imaged in STEM). Fig. 3B (higher magnification) was taken at an exceptionally thin spot within the catalytic layer and therefore allows to identify individual nanoparticles which appear adjacent to each other or stacked on top of each other. Both micrographs strongly resemble previously published STEM of PDA-linked Pt NP networks synthesized on carbon-coated TEM grids for which STEM tomography had demonstrated that they are composed of discrete Pt NP with inter-particle distances in the range of NP diameters.^4^ The STEM data thereby indicate that the procedures described in the experimental section produced a continuous layer of a microporous PDA-linked Pt NP network also on the technically relevant SiN substrate, as desired. Note that the average thickness of the catalytic layer can be estimated as 2 μm as detailed in the ESI.† For comparison, Fig. 3C and D show STEM of plain Pt NP deposited on a SiN sensor membrane and reveal structural differences from PDA-linked Pt NP. The overview (Fig. 3C) reveals a grainy structure with an overall inhomogenous distribution of Pt on the membrane. Even at higher magnification, sites where individual Pt NP could be resolved were very rare. Instead, coherent Pt structures were predominant, indicating agglomeration and partial sintering of plain Pt NP after deposition (see Fig. 3D). Note that the same total amount of Pt was deposited on both samples, *i.e.*, the sensor with the PDA-linked Pt NP catalyst and the sensor with the plain Pt NP catalyst.

**Fig. 4:**
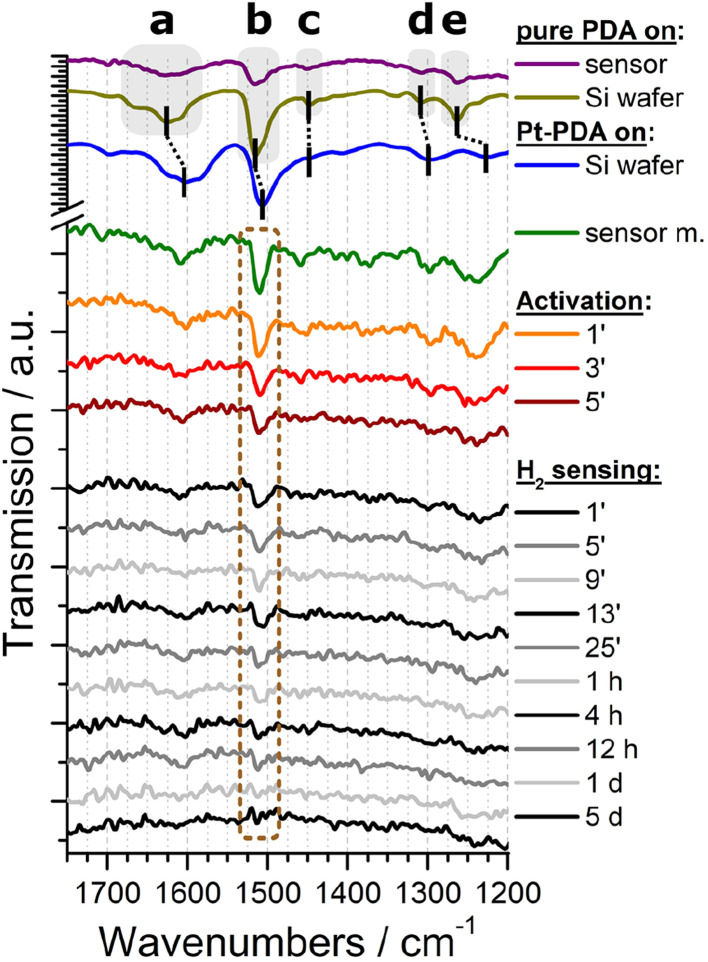
Top section: FTIR spectra of pure PDA on a sensor membrane (purple), pure PDA on a Si wafer (olive), and Pt-PDA on a Si wafer (blue). Bottom section: *In-operando* FTIR spectra of Pt-PDA on a sensor membrane before (green) and during catalyst activation (orange, red, brown) and during H_2_ gas sensing operation (grey and black). Labels represent time stamps of the spectra in minutes, hours and days during catalyst activation and sensor operation, resp.

Both types of sensor samples were put underneath the procedures for catalyst activation and then immediately underwent a long-term stability test of their sensor performance at an idle operation temperature of 70 °C and with a constant flow of 1.5 vol% H_2_ in synthetic air through the reactor module, as detailed in the experimental section. *In operando* FTIR spectra were recorded automatically at time intervals of 2 min during activation and 4 min during sensor operation, respectively. As background signal, the spectrum obtained from a sensor membrane without catalyst was subtracted. For plain Pt NP catalysts, no vibrational bands could be identified in the FTIR spectra (see ESI Fig. SI 4†). An exemplary selection of FTIR spectra obtained from a sensor membrane covered by a catalytic layer of PDA-linked Pt NP is shown in the bottom section of Fig. 4 (shown are a spectrum before activation, three consecutive spectra obtained during activation and ten spectra at varying time intervals during a total of 5 days of H_2_ gas sensing operation). For comparison, also spectra of a sensor membrane carrying pure PDA as well as of pure PDA and PDA-linked Pt NP on a Si wafer are depicted in the top section of Fig. 4. The latter two spectra exhibit a superior signal-to-noise ratio because they were recorded as references to the *in-operando* spectra from larger samples and without the reactor module.

Both FTIR spectra of pure PDA, *i.e.*, the reference spectrum of PDA on Si (olive curve in Fig. 4) as well as the spectrum of PDA on a sensor membrane within the reactor module (purple), exhibit all characteristic vibrational bands of an aromatic amine: The most prominent bands can be ascribed to the δ(NH) scissoring mode at 1628 cm^−1^ (a), the *ν*(CC) band at 1516 cm^−1^ (b), and the *ν*(CN) stretching mode at 1263 cm^−1^ (e).^4,13^ In addition, both spectra show weak FTIR signals at 1451 cm^−1^ (c) and at 1308 cm^−1^ (d) which are fingerprints of two further *ν*(CC) vibrations of PDA.^4,13^

In comparison, the reference spectrum of Pt-PDA on Si wafer (blue curve in Fig. 4) illustrates the redshift of PDA-related bands that are expected to be observed for PDA-linked Pt NP networks. A relatively strong redshift of the amino-related bands, in particular of the δ(NH) band (a) by 19 cm^−1^, and a weaker shift of the *ν*(CC) band (b) by 5 cm^−1^ can be ascribed to N-dative bonding of the NH_2_ groups to Pt and provide evidence for the formation of PDA crosslinks between Pt NP.^4,14^ Despite the comparatively low signal-to-noise ratio of the FTIR spectrum obtained from freshly prepared Pt-PDA on a sensor membrane in the reactor module (green curve) it exhibits all characteristic vibrational bands of PDA and in particular the bands a, b and e at positions in close agreement with the reference spectrum of PDA-linked Pt NP networks on Si wafer. On the one hand, the FTIR data of freshly prepared sensor samples thereby provide evidence for the desired formation of PDA-linked Pt NP catalysts on the SiN sensor membranes, in corroboration with the STEM data. On the other hand, the data show that the experimental setup for *in operando* FTIR spectroscopy detailed in the experimental section was sufficiently sensitive to detect the technically relevant minute amounts (≈0.5 μg) of PDA used for the hydrogen gas microsensor design.

The series of *in-operando* FTIR spectra shown in the bottom section of Fig. 4 reveals at first sight a continuously progressing loss of intensity of all PDA-related vibrational bands in the Pt-PDA sensor sample during catalyst activation and subsequent sensor operation. Neither shifts of band positions or relative intensities nor the formation of new bands were observed. Therefore, the band intensities were considered as being proportional to the density of PDA molecules in the Pt-PDA network to a good approximation. To quantify the loss of PDA during activation and sensor operation, the integral intensity of the most intense FTIR band (*ν*(CC) band b at 1511 cm^−1^) was evaluated. For that purpose, each FTIR spectrum was integrated in the range 1535–1479 cm^−1^. To account for varying background and noise levels of the spectra, an individual base line for each spectrum was subtracted beforehand. This was defined by two interpolation points which were obtained by averaging short ranges of the respective FTIR spectrum to the left and to the right of the *ν*(CC) band, namely the ranges 1535–1520 cm^−1^ and 1489–1479 cm^−1^, respectively. To compensate for the poor signal-to-noise ratio of single *in operando* FTIR spectra obtained during sensor operation, the *ν*(CC) band integrals of three consecutive spectra were averaged.

**Fig. 5:**
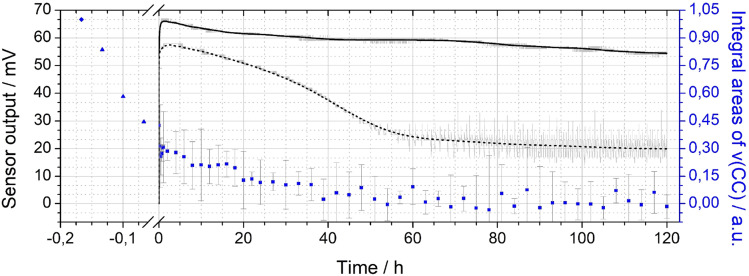
Double-*y* plot showing the sensor output (left axis) for Pt-PDA (black solid curve) and plain Pt NP (black dashed curve) catalysts during 5 days of sensor operation (raw data in grey) as well as integral values (blue data points) of the *ν*(CC) band of PDA in FTIR (right axis). Data points before sensor operation (at *t* < 0) obtained after Pt-PDA synthesis and during catalyst activation, see text for details.

The integral *ν*(CC) band intensities obtained during activation and operation of a sensor with Pt-PDA catalyst are shown *versus* total time (*t*) in Fig. 5 (blue data points), normalized to the initial *ν*(CC) band integral of the freshly prepared sample before activation. Note that the origin of the *t* axis was chosen at the start of the sensor operation after activation. (Error bars are based on the empirical standard deviation of the averaged integrals of three consecutive spectra and represent the 90% confidence interval of Student's t-distribution for small samples.) Concomitantly, Fig. 5 shows the output Δ*U* of the sensor with Pt-PDA catalyst (solid line) and, for comparison, the sensor output of a sensor with plain Pt NP as catalyst (dotted line).

With respect to the *ν*(CC) intensity as a function of time, a large difference between the loss rates during activation (catalyst temperature *T* = 180 °C) and during sensor operation (*T* = 120 °C) becomes evident at first glance. Overall, after 60 h of sensor operation the *ν*(CC) intensity data show no significant deviation from zero within the experimental error. Numerical fits to the data and a logarithmic plot of the *ν*(CC) intensity as a function of *t* (see Fig. SI 5 in the ESI†) reveal that the integral *ν*(CC) band intensity is well approximated as simply proportional to exp(−*k* × *t*) with a rate constant *k* of about ln(2)/(5 min) during activation and of about ln(2)/(20 h) during sensor operation, respectively. Assuming that the *ν*(CC) intensity is representative of the PDA density in the Pt NP network, the data therefore indicate that about half of the initial amount of PDA in the sample was probably lost due to decomposition and desorption after 5 min of activation and that the PDA density was further reduced by factors of about 1/2 every 20 h of sensor operation. Consequently, after activation and 5 days of sensor operation less than 1% of the initial amount of PDA should have remained in the catalyst.

Despite the almost complete loss of PDA in the Pt-PDA catalyst, the sensor output remained fairly stable over 5 days of operation. It decreased only by 18% from its maximum value which was reached within the first hour of operation. In contrast, the sensor with pure Pt NP as catalyst showed a pronounced decay of its output to about 1/3 of its maximum value within the first two days of operation. Afterwards, it provided a rather unstable sensor signal (see the scattering of raw sensor data shown in grey in Fig. 5). While the superiority of the long-term stability of H_2_ microsensors with Pt-PDA catalysts had been observed before,^3,5,12^ it was generally assumed to be due to structural stability and intactness of the PDA-linked network of discrete Pt NP which should prevent the Pt NP from sintering and thereby maintain the catalytically active Pt surface area.^2,3^ In contradiction, the *in operando* FTIR data on Pt-PDA composition reveal that PDA-linked Pt NP networks undergo significant structural changes during sensor operation. These must be qualitatively different, however, from those of plain Pt NP catalysts as the sensor performance data demonstrate. Before presenting first data on the nanoscale structure of Pt-PDA catalysts after long-term sensor operation, possible mechanisms of PDA decomposition during activation and sensor operation will be discussed in the following.

A recent study on thermally induced decomposition of PDA-linked Pt NP networks by temperature-programmed desorption spectroscopy (TPD) in vacuum identified three different bonding configurations of PDA in freshly synthesized Pt-PDA (with 0, 1, or 2 dative NH_2_ bonds to Pt) which differed with respect to thermal stability.^6^ It was demonstrated that only the thermally most stable PDA species with two Pt bonds (double-bonded) is relevant for the structural stability of the Pt NP network. Therefore, in the present work, the maximum catalyst temperature during activation (180 °C) was kept below the onset of thermally induced decomposition of double-bonded PDA species reported at 200 °C, but above the onset of decomposition of single-bonded PDA. Consequently, some loss of PDA during catalyst activation was expected. Furthermore, one may speculate that the removal of PDA with less than two Pt bonds from the network might even be the key to catalyst activation because it should free adsorption sites on the Pt NP.

The decomposition of even double-bonded PDA from Pt-PDA during subsequent long-term sensor operation at a maximum temperature of only 120 °C was not expected. Thermogravimetry had indicated that, in plain air, thermally induced decomposition of any PDA in the NP network should set in only above 130 °C.^4^ Consequently, the continuous loss of PDA during sensor operation observed in the present study must be ascribed to promotion by H_2_. In fact, a Pt-catalyzed cleavage of C–N bonds *via* hydrogenolysis reactions, reported for the thermally induced decomposition of aniline,^15–17^ was previously found to be relevant for the decomposition of PDA in Pt-PDA, too.^6^ The major decomposition products NH_3_, aniline and benzene are highly volatile. In accordance, the *in operando* FTIR data do not show any fingerprints of residual fragments or decomposition products of PDA in the catalyst. Overall, they indicate that even at concentrations as low as 1.5 vol% H_2_ in air, hydrogenolysis significantly deteriorates the thermal stability of PDA.

**Fig. 6:**
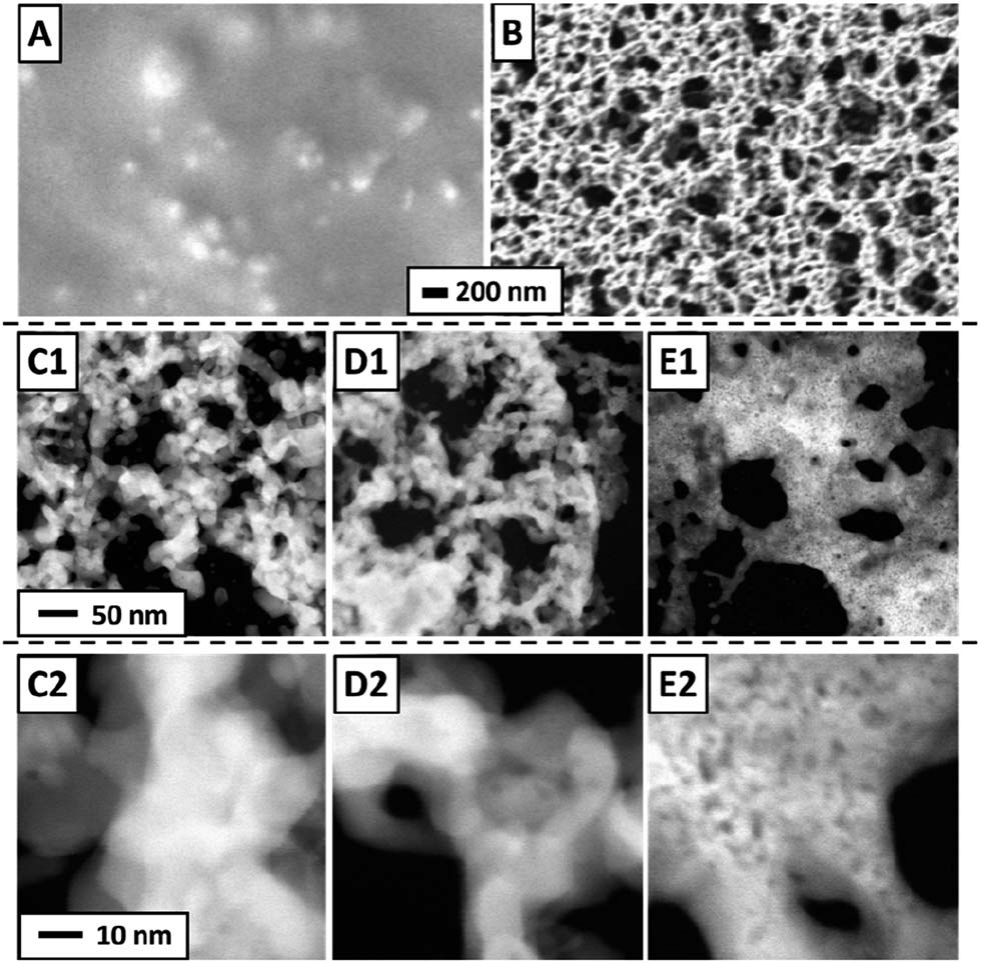
SEM of Pt-PDA on SiN membrane after deposition but before activation (A) and after 5 days of sensor operation (B); STEM of Pt-PDA after 5 days of sensor operation (C1) and (C2) and after catalyst activation (D1) and (D2); STEM of plain Pt NP on SiN membrane after 5 days of sensor operation (E1) and (E2).

Concomitantly with the decomposition of all PDA, the Pt NP network structure should significantly change. SEM and STEM micrographs acquired from sensor catalyst samples directly after preparation, after catalyst activation and after long-term sensor operation are shown in Fig. 6. On the micrometer scale, SEM of a Pt-PDA layer on a SiN membrane shows a closed and relatively smooth surface before activation (Fig. 6A) whereas it reveals a macroporous, sponge-like structure of the layer after 5 days of sensor operation (Fig. 6B). The microporous structure of freshly prepared Pt-PDA (*cf.*Fig. 3A and B) was not resolved in SEM. As Fig. 6C1 and C2 demonstrate, the catalyst structure drastically changed also on the nanometer scale during long-term sensor tests. Instead of individual Pt NP, STEM revealed mesopores with continuous Pt ligaments of a width in the range of 10–100 nm. Moreover, STEM images acquired after the activation step (Fig. 6D1 and D2) were similar to those obtained after 5 days of sensor operation, *i.e.*, the PDA-linked NP network had already collapsed even before sensor operation started. Note that measurements of the thickness of the Pt-PDA layer after 5 days of sensor operation *via* SEM at random sites along few available breaking edges yielded values in the range 1–2.5 μm, *i.e.*, in the range of the estimated average thickness of the freshly prepared Pt-PDA layer.

For comparison, STEM micrographs of a plain Pt NP catalyst on a SiN sensor membrane acquired after 5 days of sensor operation are shown in Fig. 6E1 and E2. They demonstrate a significant structural difference between pure Pt NP and Pt-PDA after catalysis: plain Pt NP appeared to have sintered into a more compact layer, whereas the Pt-PDA-based catalysts maintained a mesoporous structure, although the PDA-linked Pt NP network had collapsed.

**Fig. 7:**
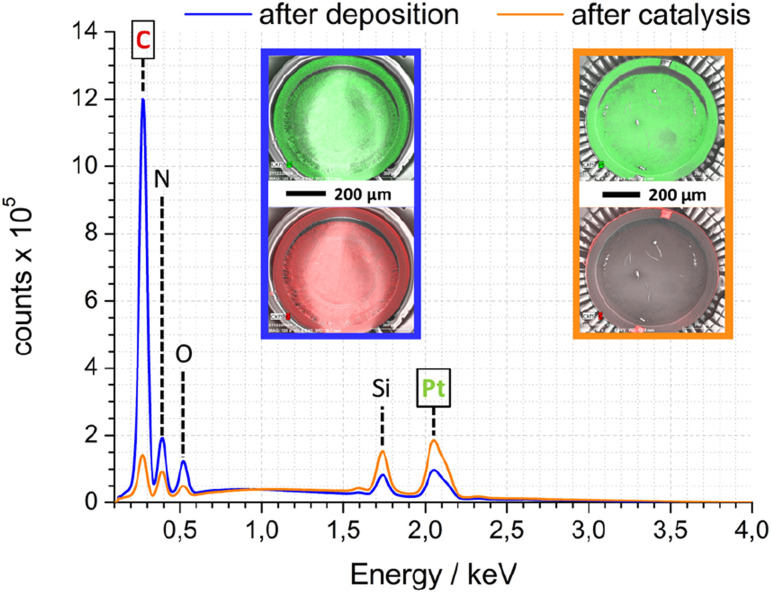
SEM-EDX spectra of Pt-PDA-based catalyst on SiN sensor membrane after deposition (blue) and after 5 days of sensor operation (orange); insets show EDX signal intensity maps for Pt (green – top) and C (red – bottom) as overlays over corresp. SEM images (greyscale).

As complement to FTIR and electron microscopy data, EDX was employed to analyse the elemental composition of Pt-PDA-based catalysts. Fig. 7 shows EDX spectra obtained while imaging an entire SiN sensor membrane with a Pt-PDA catalyst by SEM. Two spectra are shown, one obtained directly after Pt-PDA deposition, *i.e.*, before catalyst activation (blue curve), and one obtained after 5 days of sensor operation (orange curve). Besides Si and N, only carbon, oxygen and platinum were identified by major EDX signals as denoted in Fig. 7. After activation and sensor operation, the ratio of the C-related to the Pt-related EDX signals had decreased by a factor of about 15. The insets in Fig. 7 show EDX signal intensity maps for the spatial distribution of Pt (green) and C (red) on the SiN membrane (underlying greyscale images are the corresponding SEM data). Directly after deposition of Pt-PDA (left inset with blue frame), C and Pt showed a very similar, fairly even distribution. After 5 days of sensor operation (right inset with orange frame), Pt remained distributed homogeneously on the membrane while significant EDX intensity for C was obtained only from a small fringe around the membrane.

**Fig. 8:**
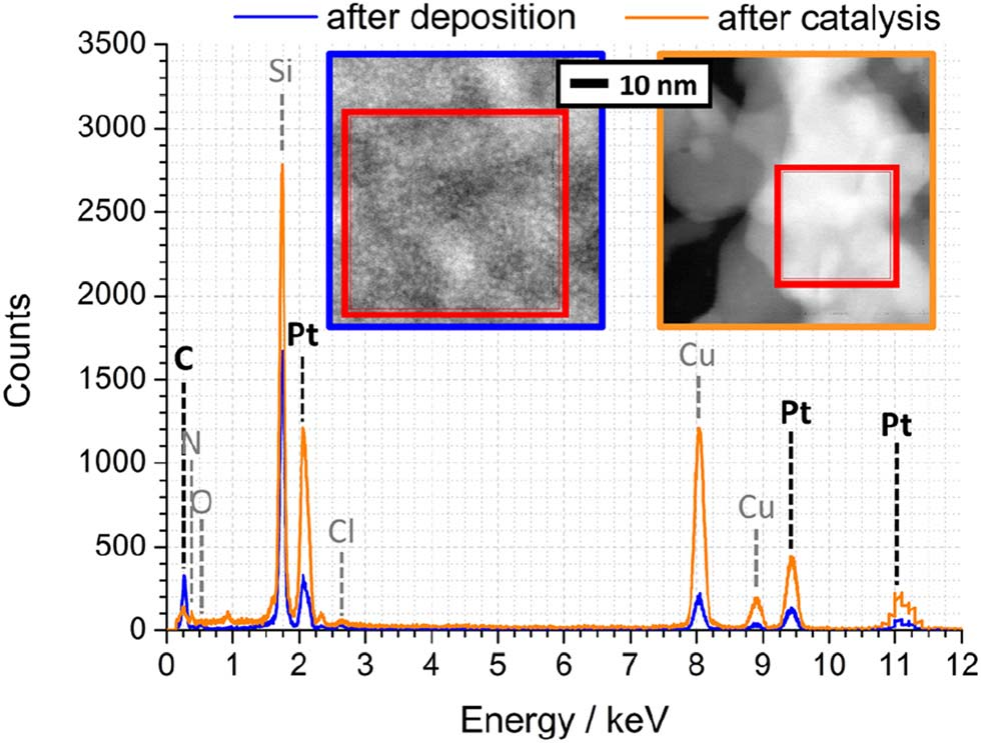
EDX spectra of Pt-PDA after synthesis (blue) and after 5 days of sensor operation (orange). Insets: STEM of corresponding nanostructures, EDX integration areas are marked.

To quantify the elemental composition of the Pt-PDA-based catalyst with a spatial resolution on the length scale of the observed mesoporosity, EDX spectra were acquired during STEM, too. Fig. 8 shows one spectrum obtained from the PDA-linked Pt NP network directly after Pt-PDA deposition (blue curve) and one from the mesoporous structure after 5 days of sensor operation (orange curve). Corresponding STEM images are shown in the insets. Therein, the sample regions from which the EDX spectra were collected are marked (red squares). Note that Cu and Cl signals in the EDX spectra, detected apart from C, N, O, Si, and Pt, can be ascribed to the TEM grid and to residual traces of the Pt NP precursor (H_2_PtCl_6_·*x*H_2_O).

A quantitative analysis of the EDX spectrum obtained from the microporous Pt NP network directly after deposition yielded an atomic ratio C : Pt of about 12 : 1 (see ESI for details of the analysis†). Since PDA was deposited in an equimolar ratio to the amount of Pt during synthesis, a C : Pt ratio of 6 : 1 would be expected if the carbon content represented only PDA (C_6_H_8_N_2_). The excess carbon is probably due to cyclohexanone residues from synthesis, also observed by TPD of freshly prepared Pt-PDA^6^ and indicated by the presence of a weak *ν*(C=O) band around 1700 cm^−1^ in FTIR (*cf.*Fig. 4, green curve).

In contrast to the Pt NP network, the ligaments of the mesoporous structure observed after catalyst activation exhibited an atomic ratio C : Pt of only about 1 : 1, confirming that not only synthesis residues of cyclohexanone but also major parts of the deposited PDA and its fragments had desorbed. However, within the experimental error of the quantitative EDX analysis, the C : Pt ratio remained the same after additional 5 days of sensor operation which indicates that the carbon of PDA decomposed during sensor operation (as revealed by FTIR) was mainly left as residue on or in the catalyst. Note that EDX data from plain SiN membrane area in between catalyst ligaments showed only traces of C, and an EDX analysis of plain Pt NP on a sensor membrane yielded an atomic ratio C : Pt of only 1 : 10 (see ESI Fig. SI10 and SI11†). Consequently, the amount of carbon detected in the ligaments of the mesoporous structure can be ascribed to small but significant residues from PDA. It is nearby to assume that these residues stabilize the mesoporous, sponge-like Pt nanostructure. Furthermore, their presence indicates that in addition to hydrogenolysis reactions with solely volatile products (benzene, aniline, NH_3_),^6^ there must be at least one other relevant route of thermally induced PDA decomposition, probably similar to that identified by Huang *et al.* for monofunctional aromatic and cycloaliphatic amines adsorbed on Ni surfaces which formed partially dehydrated polymeric layers and carbides.^15,16,18^ Besides PDA, other bifunctional ligands were also used for Pt NP network synthesis in the present study (ligands BEN, DAN, DATER, and DACH as detailed in the experimental section). In particular Pt-BEN and Pt-DAN catalysts showed results very similar to PDA-linked NP networks regarding the evolution of morphology, composition and performance during catalyst activation and long-term sensor operation (see ESI for details†).

## 4. Conclusions

Combining electron microscopy, EDX and FTIR spectroscopy, the evolution of the structure of diamine-linked Pt NP network catalysts in hydrogen gas microsensors was investigated during catalyst activation and sensor operation. A novel experimental setup for *in operando* FTIR spectroscopy at working microsensors was employed which, for the first time, allowed for quantifying rates of thermally induced decomposition of the diamine links during sensor operation. Exemplarily shown for PDA, aromatic diamines in the network could be identified and quantified *via* a characteristic CC stretching mode in FTIR even from minute amounts of PDA in the microgram range. In contrast to findings of a previous thermogravimetric analysis in air, thermally induced decomposition of PDA was observed at temperatures as low as 120 °C which indicates that the presence of hydrogen in the ambient atmosphere promotes PDA decomposition, presumably *via* Pt-catalyzed hydrogenolysis reactions. About 50% of the PDA used for synthesis of the network already decomposed during catalyst activation, and the remaining PDA fully decomposed during 5 days of continuous sensor operation. While one may conceive that complete decomposition of the remaining PDA into volatile products should free more Pt surface sites, *i.e.*, could even increase the catalyst activity to some extent, at the end the Pt NP network is expected to be prone to sintering without PDA which should, overall, quench the catalyst activity in a fashion similar to that observed for plain Pt NP catalysts. Still, the sensor demonstrated a steadily high sensitivity to hydrogen during the long-term test, far superior to that of plain Pt NP catalysts, but apparently uncorrelated with the stability of the PDA links in the network.

Electron microscopy revealed that the synthesized network structure of discrete Pt NP inter-linked by PDA molecules was transformed into a sponge-like nanostructure with continuous Pt ligaments already during catalyst activation. These findings disprove assumptions of previous work in which stability of the diamine ligands and the microporous network structure, composed of ligand-linked, discrete Pt NP, was regarded as basis for the observed superior sensor performance. While plain Pt NP sintered and formed a compact layer during sensor operation, the Pt-PDA-based catalysts maintained a porous structure with a high surface-to-volume ratio, probably stabilized by carbon residues from the PDA. The exact chemical nature of these residues was not resolved in the present study and remains to be clarified. If it was similar to previously observed carbon-based layers or carbides that were left over from thermally induced decomposition of self-assembled aniline layers, the residual structure of the Pt-PDA-based catalysts should be stable up to temperatures as high as 380–550 °C.^15,16^

The wide-range scale of porosity of the sponge-like Pt nanostructure revealed by electron microscopy, ranging from mesopores of few nanometers in diameter to macropores with diameters of some 100 nm, appears well suited for a heterogeneous catalyst because it should facilitate the transport of educts and products to or from Pt surface sites. Overall, porosity, thermal stability and the high catalytic activity evidenced by the hydrogen microsensor performance could render Pt-PDA-based catalysts interesting for specialized applications where a high spatial density of catalytically active sites is required. Besides for microsensors, this is given for catalytic layers within the microchannels of any form of microreactor.^19–21^ Beyond that, applications of sponge-like metal nanostructures as electrode material in, *e.g.*, electrochemical cells, supercapacitors, batteries or solar cells can be conceived.^22–24^ Compared to other fabrication or synthesis techniques, the synthesis of sponge-like Pt nanostructures *via* hydrogenolysis of Pt-PDA networks has distinct advantages. In contrast to dealloying of binary precursor metal alloys,^25,26^ it starts with liquid precursors (Pt NP in suspension and amine ligands in solution) which can be easily delivered to where a catalytic layer is required, *e.g.*, onto a sensor membrane or into microchannels of a microreactor of arbitrary geometries. Synthesis of non-supported Pt aerogels from liquid precursors was demonstrated by Eychmüller and coworkers.^27,28^ The sol–gel processing requires supercritical or freeze drying. Aerogel nanosmelting, introduced by Leventis *et al.*,^29^ may produce metal structures with tunable porosity from liquid precursors, however, only for metals which form a stable oxide.^26^ Pyrolysis of Pt NP-polymer hybrid materials at temperatures as high as ∼400–550 °C was employed by Wiesner and coworkers to produce mesoporous Pt–C composites of varying carbon content.^30^ Combustion synthesis of metal nanofoams in which complexes of tetrazol ligands and a metal ion are decomposed under inert atmospheres seems chemically related to the synthesis of sponge-like Pt nanostructures from PDA-linked Pt NP, too. Nanofoams need to be annealed at typically ∼500 °C in hydrogen to remove organic impurities.^26^

In comparison, for Pt-PDA-based catalysts, after precursor deposition a relatively short thermal treatment up to only 150 °C in presence of H_2_ turned out to be sufficient to form a porous Pt nanostructure with small carbon residues and activate the catalyst. Consequently, the production of sponge-like Pt catalyst layers *via* PDA-linked Pt NP networks as template could be the method of choice for complex geometries and delicate substrates.

## Author contributions

Daniel Loof: conceptualization, resources, investigation, formal analysis, visualization, writing – original draft. Oliver Thüringer: resources, investigation. Volkmar Zielasek: conceptualization, resources, validation, writing – review & editing, supervision. Anmona Shabnam Pranti: resources. Walter Lang: funding acquisition, writing – review & editing. Marcus Bäumer: funding acquisition, supervision.

## Conflicts of interest

The authors declare that they have no known competing financial interests or personal relationships that could have appeared to influence the work reported in this paper.

## Supplementary Material

NA-006-D3NA00955F-s001
