# Supplementary material for: In-operando FTIR study of ligand-linked Pt nanoparticle networks employed as catalysts in hydrogen gas micro sensors
Source: Nanoscale Adv. 2024 Jan 31;6(5):1436–46. doi: 10.1039/d3na00955f (PMC10898444; doi:10.1039/d3na00955f)
Supplement: NA-006-D3NA00955F-s001 [file NA-006-D3NA00955F-s001.pdf]

Electronic Supplementary Material (ESI) for Nanoscale Advances.
This journal is © The Royal Society of Chemistry 2024

Supporting Information

In-operando FTIR study of ligand-linked Pt nanoparticle networks employed as catalysts in hydrogen gas micro sensors

Daniel Loof[1], Oliver Thüringer[1], Volkmar Zielasek[1,*], Anmona Shabnam Pranti[2], Walter Lang[2], Marcus Bäumer[1]

1: University of Bremen, Institute of Applied and Physical Chemistry, Leobener Str. 6, D-28359 Bremen, Germany

2: University of Bremen, Institute for Microsensors, -actuators and -systems (IMSAS), Otto-Hahn-Allee 1, D-28359 Bremen, Germany

*: Corresponding Author

I) Details on the catalyst deposition process

Small amounts of ligand solution (0,5 µg) and Pt NP colloid (1 µg) were deposited directly on mechanically sensitive sensor membranes detailed in the main text to generate catalytic layers. For this purpose, a piezo-driven micro dispenser by Microdrop Technologies was used that allowed for the required highly accurate and contactless liquid deposition.

The dispenser head consisted of a droplet generator having a glass capillary with an inner diameter of 70 µm. This glass capillary was prone to irreversible clogging by µm-size particles. Therefore, on the one hand, all liquids used were given through a syringe filter before admission, and on the other hand, two separate nozzles of the same type (MD-K-130) were used to deposit ligand solution and Pt NP colloid, in order to prevent any mixing and reaction of both in the dispenser. In previous work, only one nozzle was used to apply the catalytic layer on sensor chips, which required time-consuming cleanup of the nozzle between the application of the ligand solution and the Pt NP colloid. In the present work, one nozzle was used exclusively for the solution of bifunctional ligands, while the other one was only loaded with Pt NP colloid.

To achieve the required accuracy of positioning both depositions, an extended dispensing stage displayed in Fig. SI 1 was used. The two dispenser nozzles were attached to a moveable holder that enabled movements in one horizontal (x) and the vertical (z) direction. The sensor chip was fixed onto a 3D-movable microscope stage with micrometer screws, which was, in particular, used for the movement in the remaining horizontal (y) direction. A macro camera (CCD camera, by Teli) having a static focal length of about 5 cm was mount close to the stage and helped to arrange the dispenser nozzle tips accurately close to the sensor membrane and to ensure precise droplet transfer and a proper dispensing volume.

To load the dispensers, the filtered ligand solution and NP colloid were each given in a clean reservoir glass bottle and tightly mounted on the nozzle holder and connected to the dispenser nozzles via flexible tubing. By applying moderate air pressure to the reservoirs via a syringe, the nozzles could be loaded with the desired dispensing liquid until the liquid spilled out of the nozzle tip. Each nozzle was

operated by one control unit (MD-E-3010) which allowed to adjust parameters for the droplet generation. For all ligand solutions as well as for Pt NP colloids in cyclohexanone, the best results for uniform droplet formation and droplet transfer to the membrane were obtained with the following parameters: Voltage = 100 V, pulse width = 200 µs and frequency = 50 Hz. Higher or lower voltages and pulse widths lead to uncontrolled scattering of the droplets or resulted in no droplet formation, respectively. The volume of each dispensed microdroplet can be estimated as $1.8*10^{-10}$ L, assuming spherical droplets with diameters of 70 µm (cf. inner diameter of glass capillary).

During sensor fabrication, a polymer cylinder (40 µm height x 660 µm diameter) was fabricated via microtechnology on each sensor membrane as container for the dispensed liquids. Exemplarily, 5 stages (a-e) of a dispensing process of 100 microdroplets of cyclohexanone into the polymer cylinder on a sensor membrane are shown in the right section of fig. SI 1. Picture **a** shows a clean sensor chip placed on the stage. In picture **b**, the nozzle tip was moved accurately over the sensor membrane. Picture **c** is taken after deposition of 100 microdroplets of pure cyclohexanone inside of the polymer cylinder. Due to surface tension, a stable hemisphere of cyclohexanone was formed on the polymer cylinder which allowed to hold 100 droplets in a reproducible manner. More applied volume eventually caused spill-over of the liquid. After the liquid was deposited, the nozzle was moved away (picture **d**), whereupon the cyclohexanone evaporated within 3 minutes (Figure **e**).

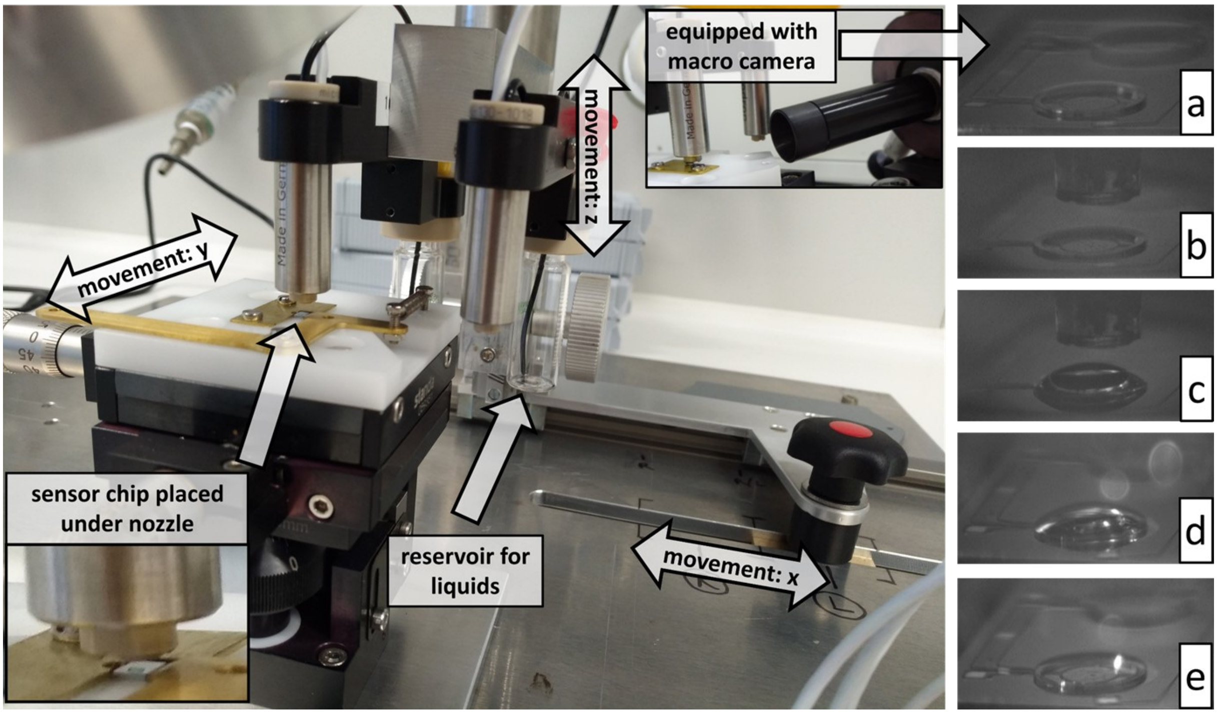

Fig. SI 1 Left: Micro dispenser stage employed for deposition of catalytic layers on sensor membranes. Right: steps of a dispensing process, in this case with pure cyclohexanone (a-e).

For deposition of catalytic layers, the two loaded dispenser nozzles were moved one after the other above the sensor membrane so that both reactants were applied consecutively. More precisely, 50 microdroplets of a freshly prepared 123 mM ligand solution in cyclohexanone were dispensed in the polymer cylinder first. Directly afterwards, 50 microdroplets of Pt NP colloid (24 g/L, 123 mM) redispersed in cyclohexanone were added which resulted in a total liquid volume of around $1.8*10^{-8}$ L with an equimolar ratio of PDA to Pt atoms. Afterwards, time was allowed for the reaction of ligand

and Pt NP and evaporation of the solvent cyclohexanone. This procedure was carried out in 4 repetitive cycles to deposit a sufficient amount of catalytic material which was detectable by the FTIR-spectrometer. The top section of Fig. SI 2 depicts three stages of one of these repetitive cycles. Due to the stability of the Pt NP redispersed in cyclohexanone, this colloid could be used for a maximum of 2 hours. After finishing 4 cycles of the dispensing process, the nozzles were thoroughly cleaned by purging several milliliters of pure acetone to completely remove the dispensing liquids. Subsequently, air was flushed through the nozzle in order to dry the capillary and avoid falsification of the concentration for any following dispensing series.

The total deposit after 4 cycles of deposition corresponded to approx. 0.9 µg of Pt and 0.5 µg of PDA which were finally crosslinked within the polymer cylinder after drying. As fig. SI 2 shows, the PDA-linked Pt NP layer was fairly homogenously distributed within the cylinder. Note that more than 4 dispensing cycles resulted in deteriorated FTIR spectra due reduced IR transparency of thicker catalytic layers. Also note that properties of the catalytic layer could be varied systematically by varying the standard dispensing procedure described above in several aspects such as type of ligand solution, type of NP colloid, total count of microdroplets, count of droplets per dispensing cycle, concentration, and ratio of Pt to ligand.

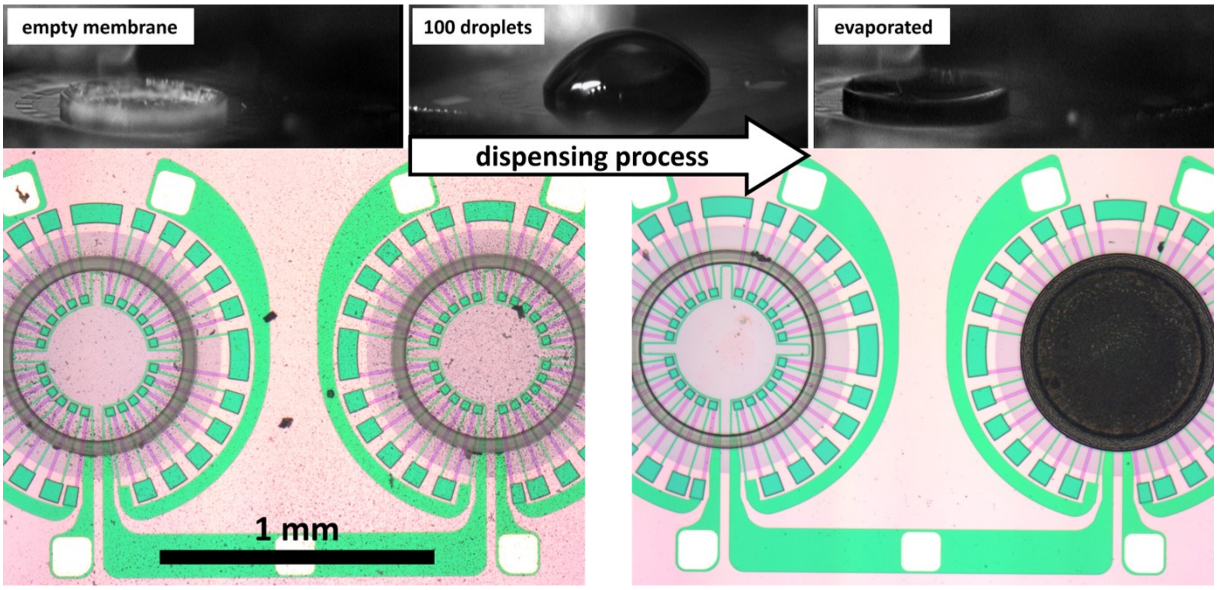

Fig. SI 2: Thermoelectric sensor before and after deposition of Pt-PDA prepared by four repetitive cycles of a standard dispensing procedure.

II) Estimate of the thickness of a catalytic layer

The thickness of catalytic layers deposited according to the procedure described above (see section I) was estimated as follows: a total volume of $3.6*10^{-8}$ L (200 droplets) of Pt NP colloid with a Pt atom concentration of 0.123 mol/L corresponds to $2.667*10^{15}$ Pt atoms on a sensor membrane. The Pt NP had diameters of ~1.5 nm which indicates that the NP are composed of 2 atomic shells [4] with a total of about 55 Pt atoms per NP [31]. Consequently, the number of Pt NP deposited on a sensor membrane can be estimated as $4.849*10^{13}$.

As determined by TEM in previous work,[4] the Pt NP within a PDA-linked network have a nearest-neighbour distance of about 1 nm and are coordinated by – on average – 6 nearest neighbours. Since this coordination corresponds to that expected for a simple cubic lattice of Pt NP, the average volume per Pt NP within the catalytic layer is estimated in analogy as $(2.5\ nm)^3=15.625*10^{-27}\ m^3$ (with 2.5 nm corresponding to NP diameter plus average nearest neighbour distance). Given the number of Pt NP deposited on a sensor membrane determined above, the total volume of the ligand-linked network on a sensor membrane can therefore be estimated as $7.577*10^{-13}\ m^3$. Assuming the formation of a homogenously thick catalytic layer within the polymer cylinder (660 µm diameter, i.e., $3{,}421*10^{-7}\ m^2$ base area) on the sensor membrane, its thickness should correspond to $2.2*10^{-6}$ m, i.e., to about 2 µm.

III) *In-operando* FTIR data of a sensor with plain Pt NP catalyst

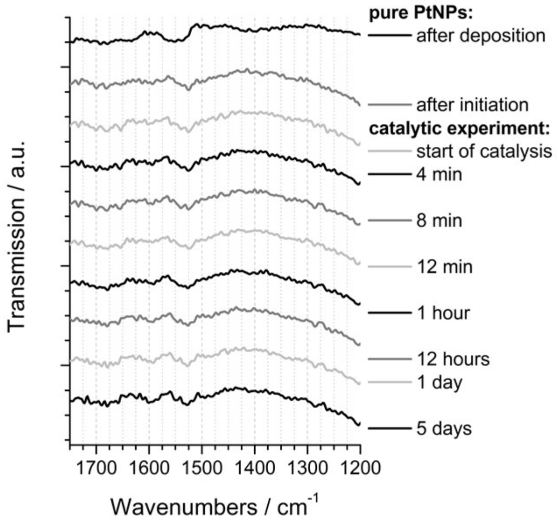

Fig. SI 3: *In-operando* FTIR spectra of pure Pt NP on sensor membrane after deposition, after activation and during catalytic H_2 gas sensing.

IV) Logarithmic plot of ν(CC) intensity vs. time and numerical fits to the data

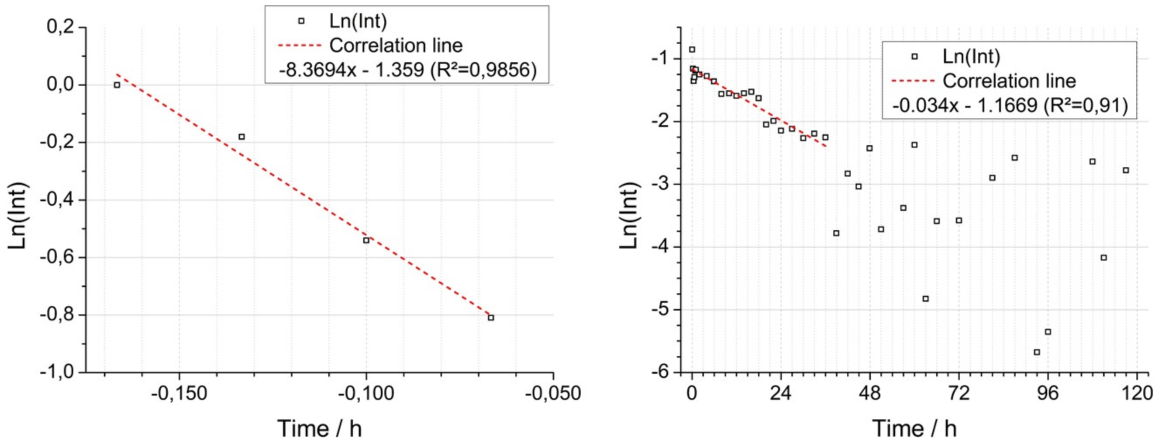

Fig. SI 4: *In-operando* FTIR data of Pt-PDA presented as logarithmic plots of the integral intensity of the ν(CC) band versus time in hours during activation (left) and during sensor operation (right)

V) Quantification of STEM-EDX data

Tab. S1: Elemental quantification from EDX by TIA software in atom% and calculated C(K)-to-Pt(L) ratios for blank SiN sensor membrane as well as for SiN sensor membranes with ligand-linked Pt NP network or Pt NP catalysts after deposition, after activation, and after catalysis

Sample	C(K)	N(K)	O(K)	Si(K)	Cl(K)	Pt(L)	C(K) : Pt(L)	note
**blank SiN sensor membrane**								
with C-Film	2	14	(0.2)	83	(0.4)	-	-	underlying carbon layer (of the TEM grid) not detectable
without C-Film	3	14	0	82	(0.4)	-	-	
**after deposition**								
Pt-PDA	47	3	4	42	(0.5)	4	11.8	before catalysis
Pt NP	4	13	2	79	(0.3)	1	4.0	before catalysis
**after the activation step**								
Pt-PDA	23	(0.1)	(0.3)	49	(0.2)	28	0.8	fully activated
**after catalysis**								
Pt-PDA	13	9	2	64	(0.1)	12	1.1	determined on catalyst
Pt-PDA	2	6	(0.6)	91	(0.5)	0	-	determined on SiN
PtNPs	2	7	0	75	(0.1)	16	0.1	determined on catalyst
PtNPs	2	6	0	92	(0.6)	0	-	determined on SiN
Pt-DACH	8	6	1	75	(0.3)	9	0.9	determined on catalyst
Pt-DACH	2	4	0	94	(0.5)	0	-	determined on SiN
Pt-BEN	14	5	3	57	(0.4)	22	0.6	determined on catalyst
Pt-BEN	2	1	0	97	0	0	-	determined on SiN
Pt-DAN	16	8	1	55	(0.2)	20	0.8	determined on catalyst
Pt-DAN	4	10	1	85	(0.1)	0	-	determined on SiN
Pt-DATER	5	4	1	67	(0.1)	21	0.2	determined on catalyst

Tab. S1 summarizes the results of EDX quantification (based on K- or L-lines) with respect to all relevant elements (C, N, O, Si, Cl, and Pt). All EDX quantifications, in particular the numbers for light elements such as C, N, and O should be considered as estimates because samples were analyzed without further treatment and without taking into account variations of sample texture. An extensive sample preparation with an internal standard would have been necessary for optimal quantification by EDX. [32],[33] In addition to the EDX data presented in the main publication, the STEM/EDX data presented in the following figs. SI 5-15 were used for the elemental quantification summarized in table S1. Note that the carbon film of the TEM grid underneath 500 nm thick SiN membrane samples did not contribute significantly to the C(K) EDX signal. The EDX spectra were essentially the same, whether the carbon film was under the membrane or not (see fig. SI 5 -2 and 6 -1).

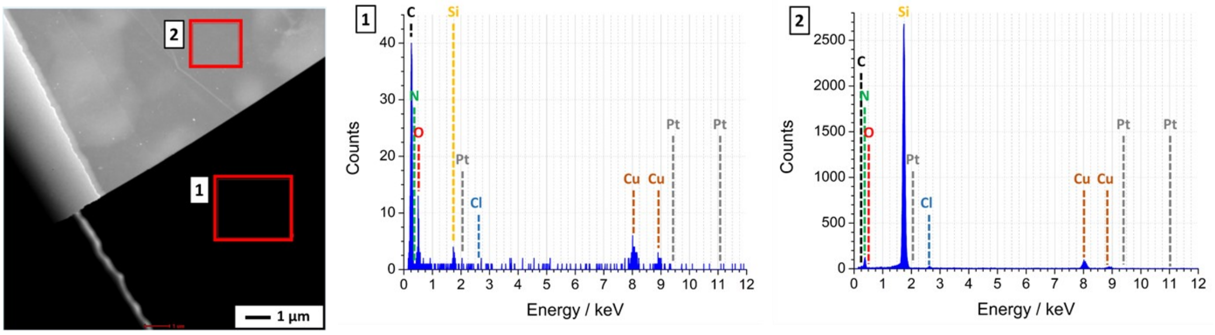

Fig. SI 5: STEM EDX data of **1**) carbon film of the TEM grid and **2**) clean SiN sensor membrane with underlying carbon film of the TEM grid.

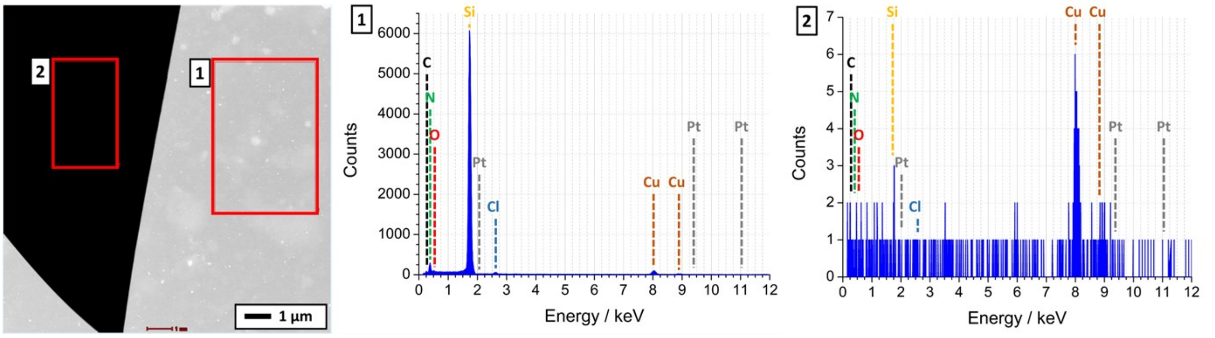

Fig. SI 6: STEM EDX data of **1**) clean SiN sensor membrane without any underlying carbon film and **2**) empty space.

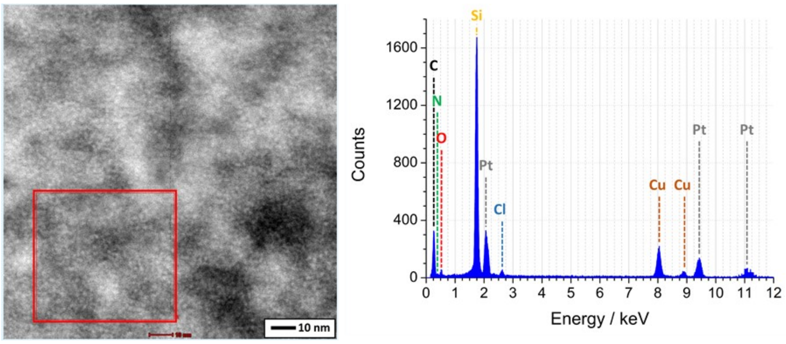

Fig. SI 7: STEM EDX data of Pt-PDA after deposition on a sensor membrane.

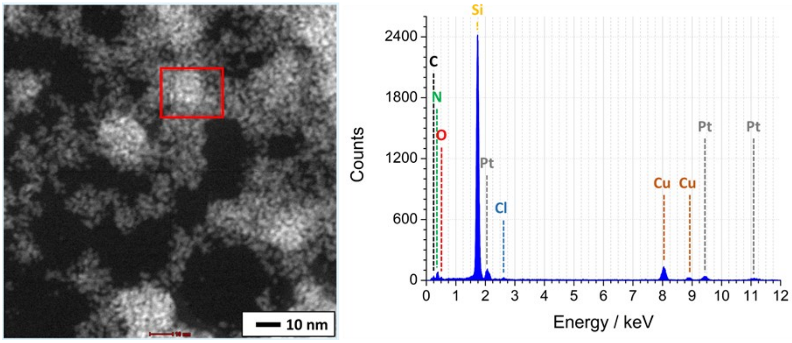

Fig. SI 8: STEM EDX data of pure Pt NP after deposition on a sensor membrane.

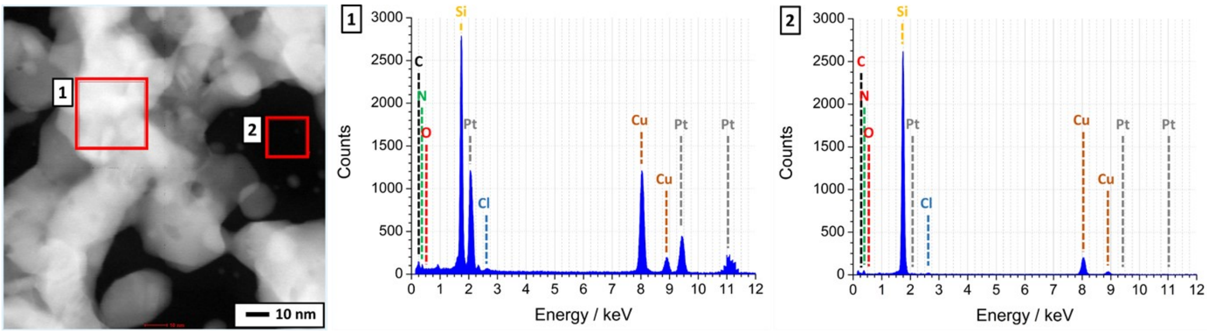

Fig. SI 9: STEM EDX data of Pt-PDA on sensor membrane after five days of sensor operation. EDX spectra acquired **1**) directly on the catalyst and **2**) on blank SiN membrane.

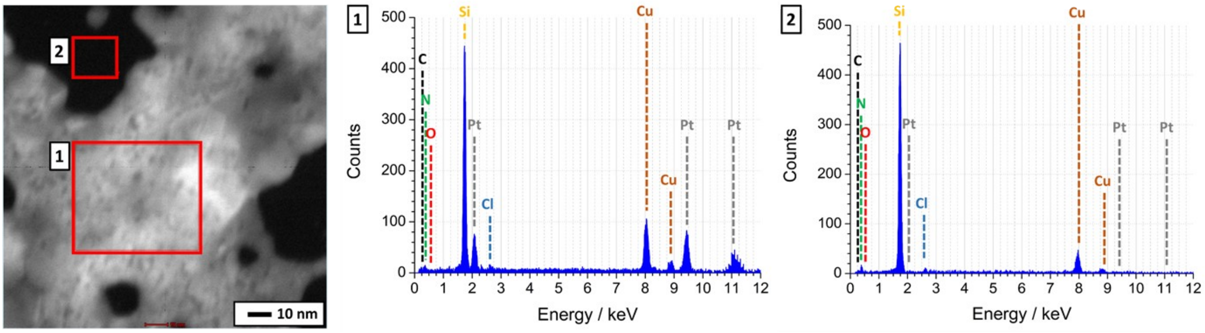

Fig. SI 10: STEM EDX data of pure Pt NP on sensor membrane after five days of sensor operation. EDX spectra acquired **1**) directly on the catalyst and **2**) on blank SiN membrane.

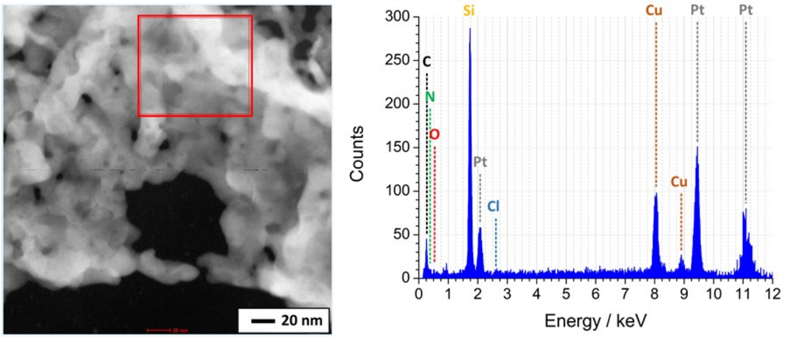

Fig. SI 11: STEM EDX data of Pt-PDA on sensor membrane after activation.

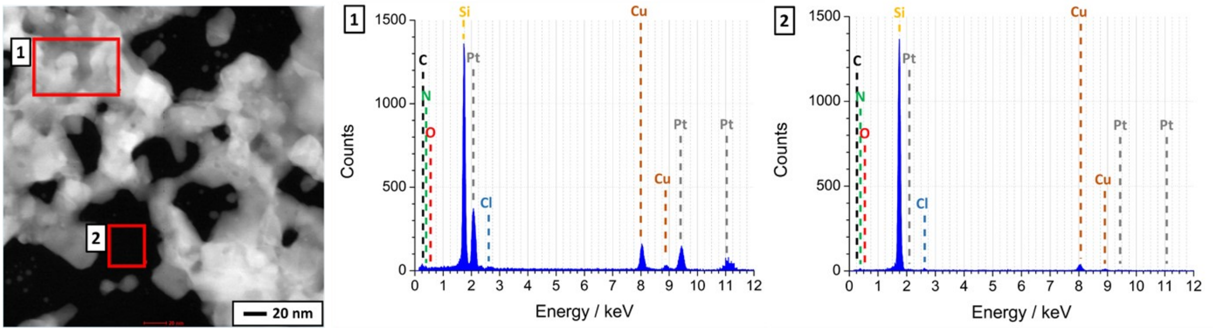

Fig. SI 12: STEM EDX data of Pt-DACH on sensor membrane after 5 days of sensor operation. EDX spectra acquired **1**) directly on the catalyst and **2**) on SiN membrane.

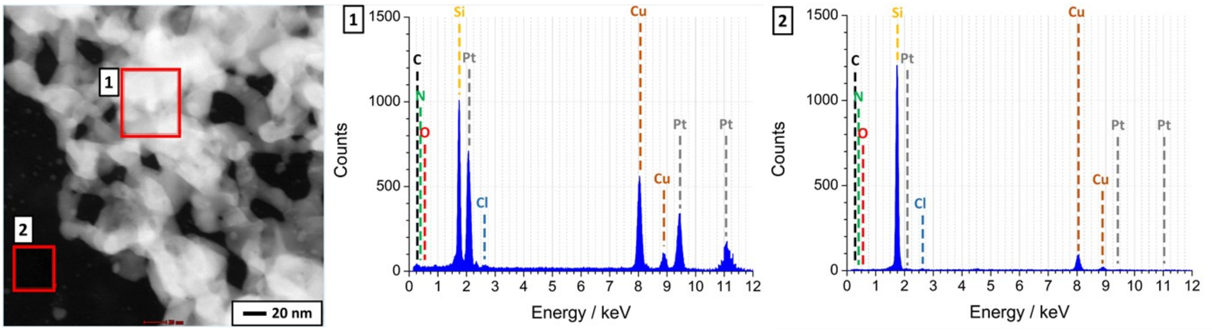

Fig. SI 13: STEM EDX data of Pt-BEN on sensor membrane after 5 days of sensor operation. EDX spectra acquired **1**) directly on the catalyst and **2**) on SiN membrane.

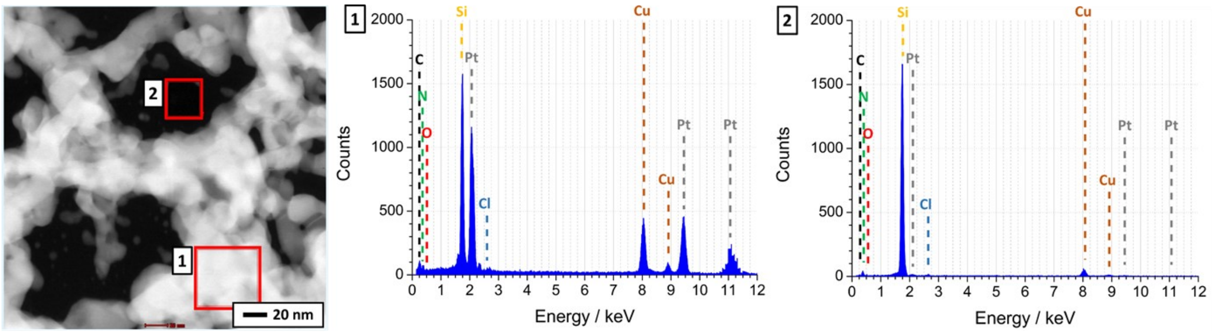

Fig. SI 14: STEM EDX data of Pt-DAN on sensor membrane after 5 days of sensor operation. EDX spectra acquired **1**) directly on the catalyst and **2**) on SiN membrane.

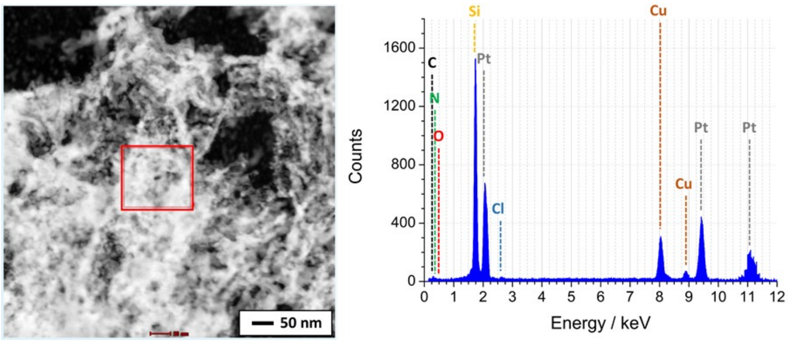

Fig. SI 15: STEM EDX data of Pt-DATER on sensor membrane after 5 days of sensor operation.

VI) *In-operando* FTIR and sensor data of Pt NP networks with DACH, BEN, DAN, and DATER

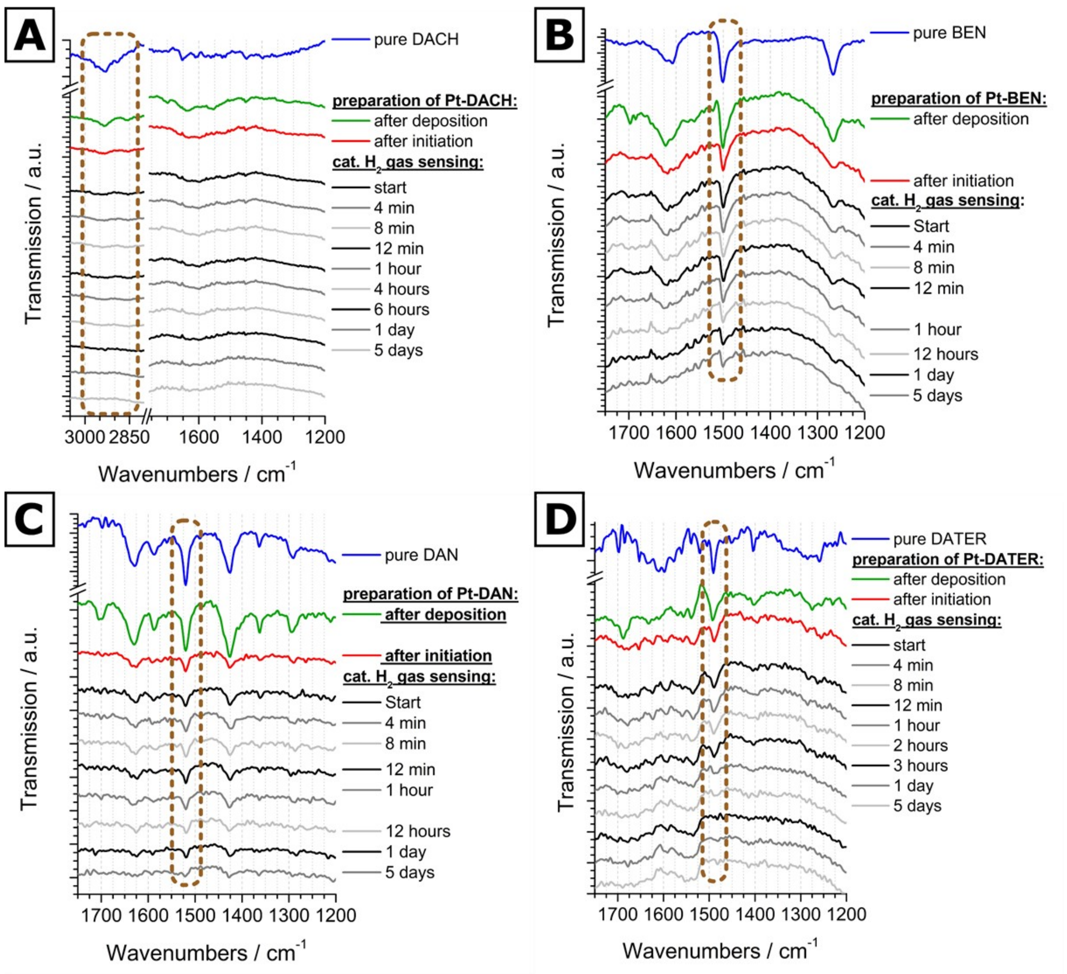

Fig. SI 16: *In-operando* FTIR spectra of **A**) Pt-DACH, **B**) Pt-BEN, **C**) Pt-DAN, and **D**) Pt-DATER on sensor membrane during 5 days of catalytic H_2 gas sensing. The brown frames highlight the most prominent bend.

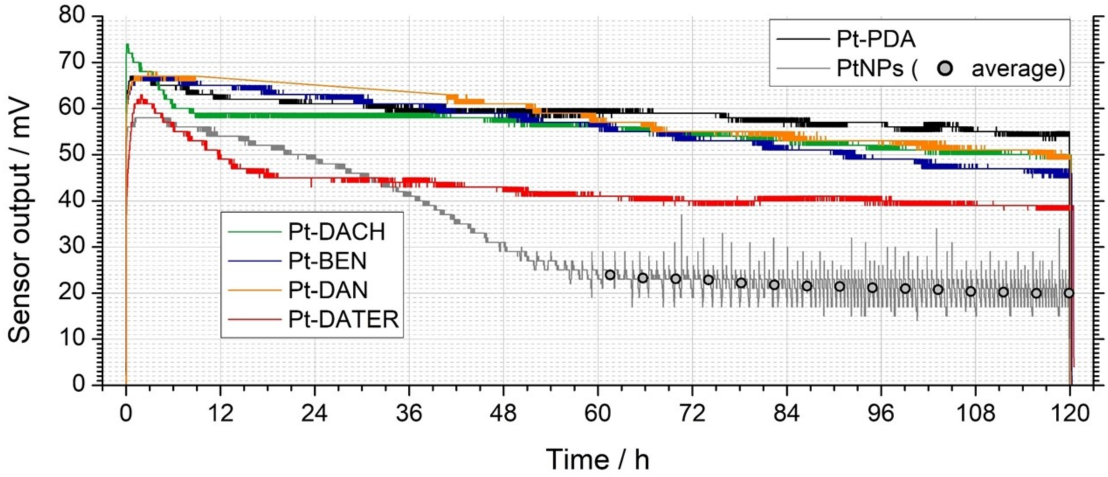

Fig SI 17: Sensor output (raw data) in mV of Pt-DACH, Pt-BEN, Pt-DAN, and Pt-DATER during 5 days of sensor operation. For comparison, the results from Pt-PDA and plain Pt NP catalysts are also shown.

References

[1] – [30] see main article

[31] T. Mori, T. Hegmann, “Determining the composition of gold nanoparticles: a compilation of shapes, sizes, and calculations using geometric considerations, ” J. Nanopart Res, vol 18, no. 10, p. 295, 2016.

[32] Dale E. Newbury, Nicholas W. M. Ritchie, “Performing elemental microanalysis with high accuracy and high precision by scanning electron microscopy/silicon drift detector energy-dispersive X-ray spectrometry (SEM/SDD-EDS)”, J. Mater. Sci., vol. 50, no. 2, pp. 493–518, 2015.

[33] D. E. Newbury and N. W. M. Ritchie, “Quantitative Electron-Excited X-Ray Microanalysis of Borides, Carbides, Nitrides, Oxides, and Fluorides with Scanning Electron Microscopy/Silicon Drift Detector Energy-Dispersive Spectrometry (SEM/SDD-EDS) and NIST DTSA-II”, Microsc. Microanal., vol. 21, no. 5, pp. 1327–1340, 2015.
